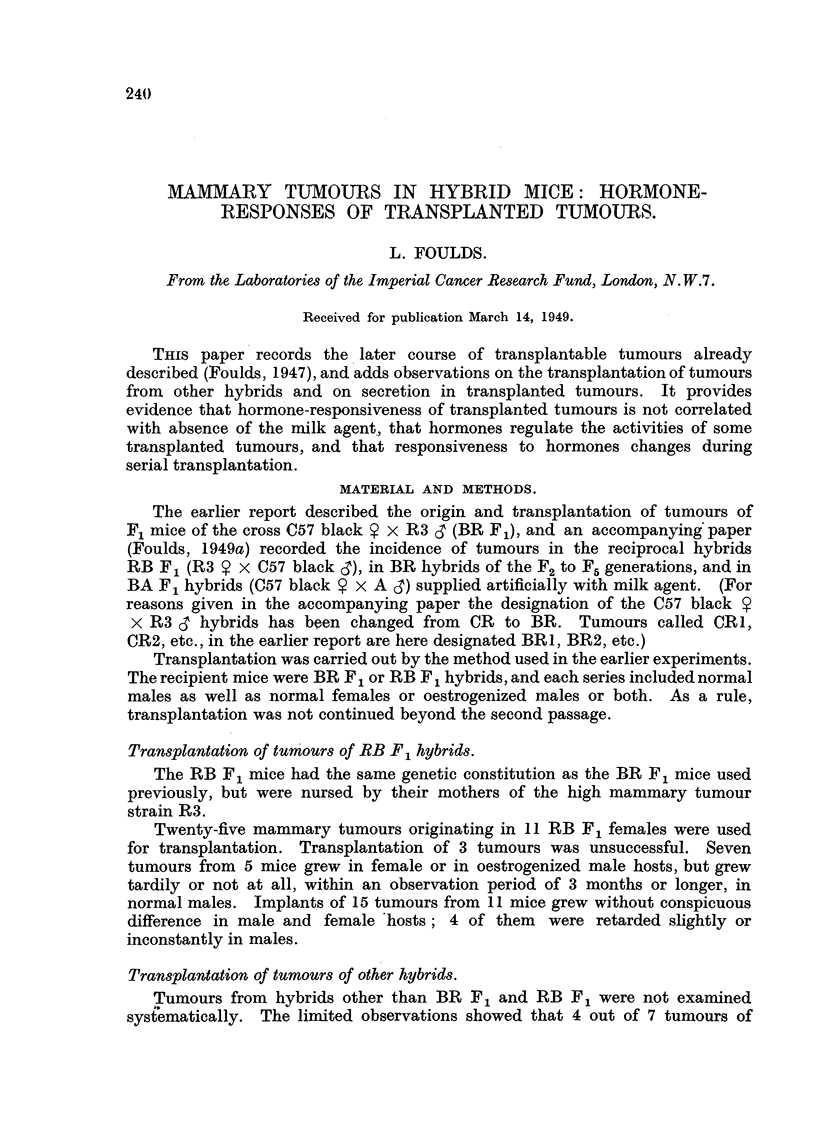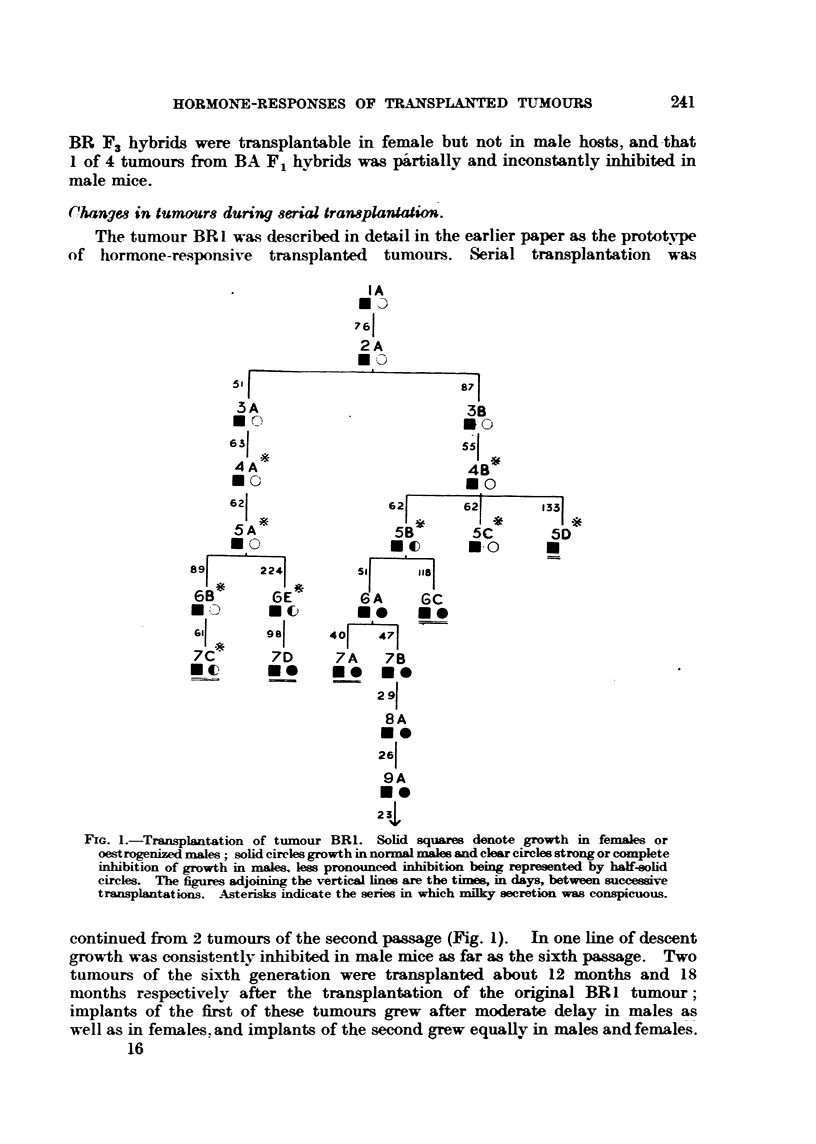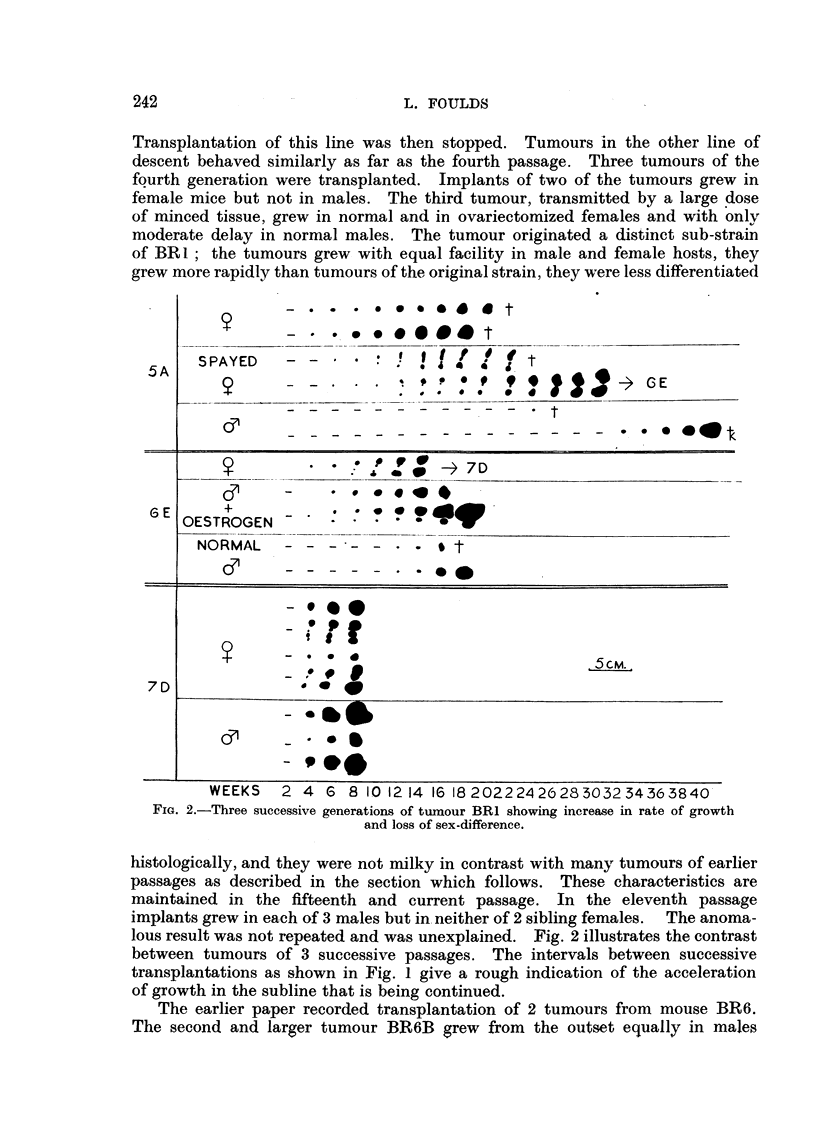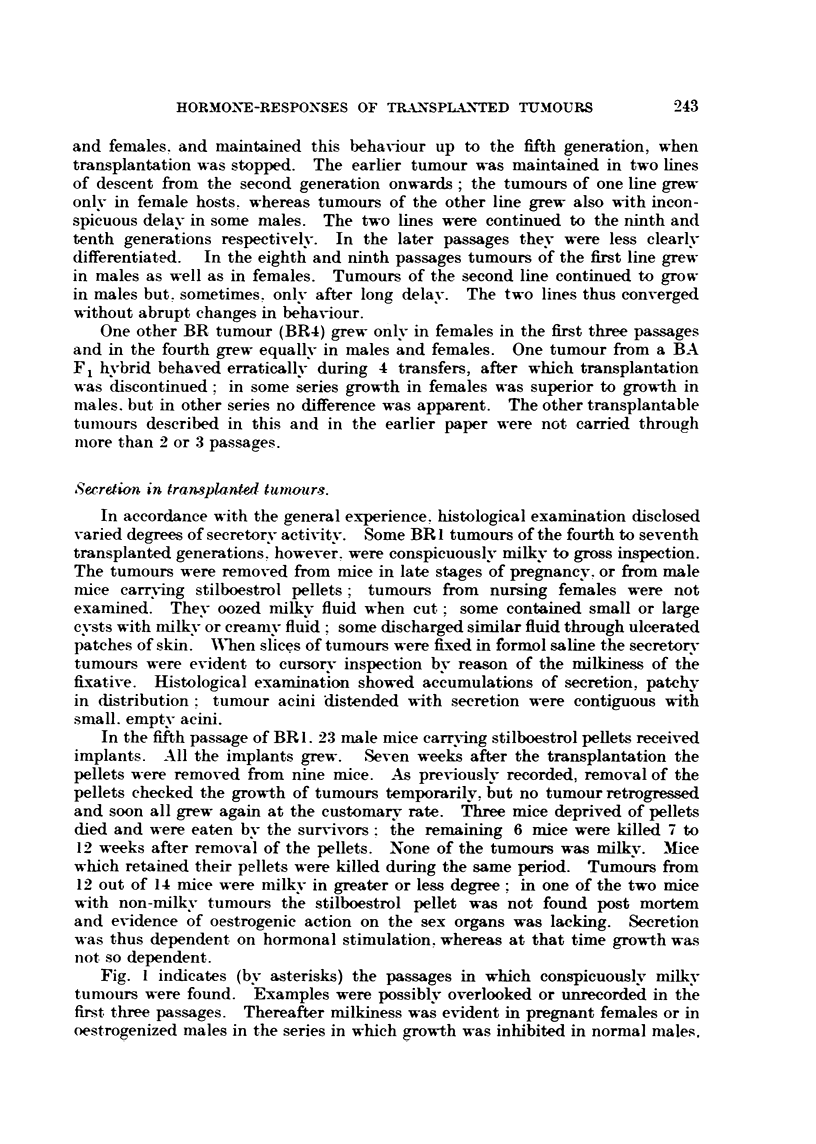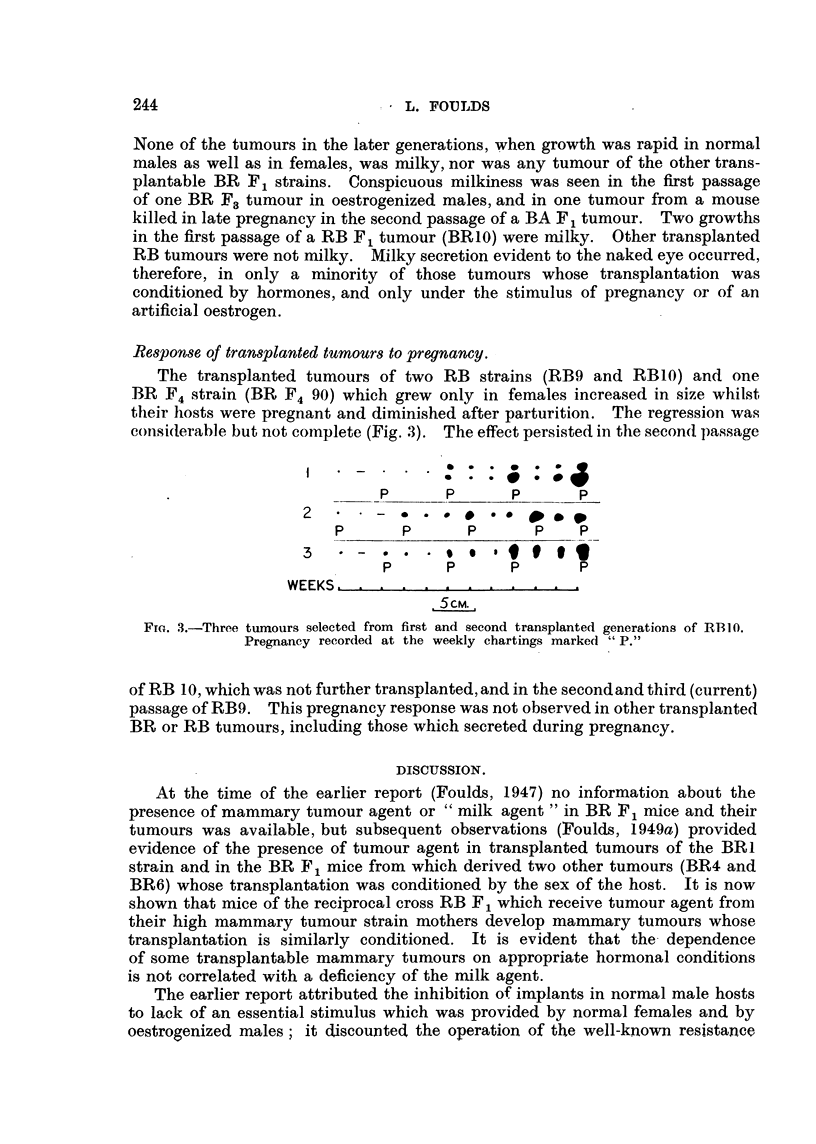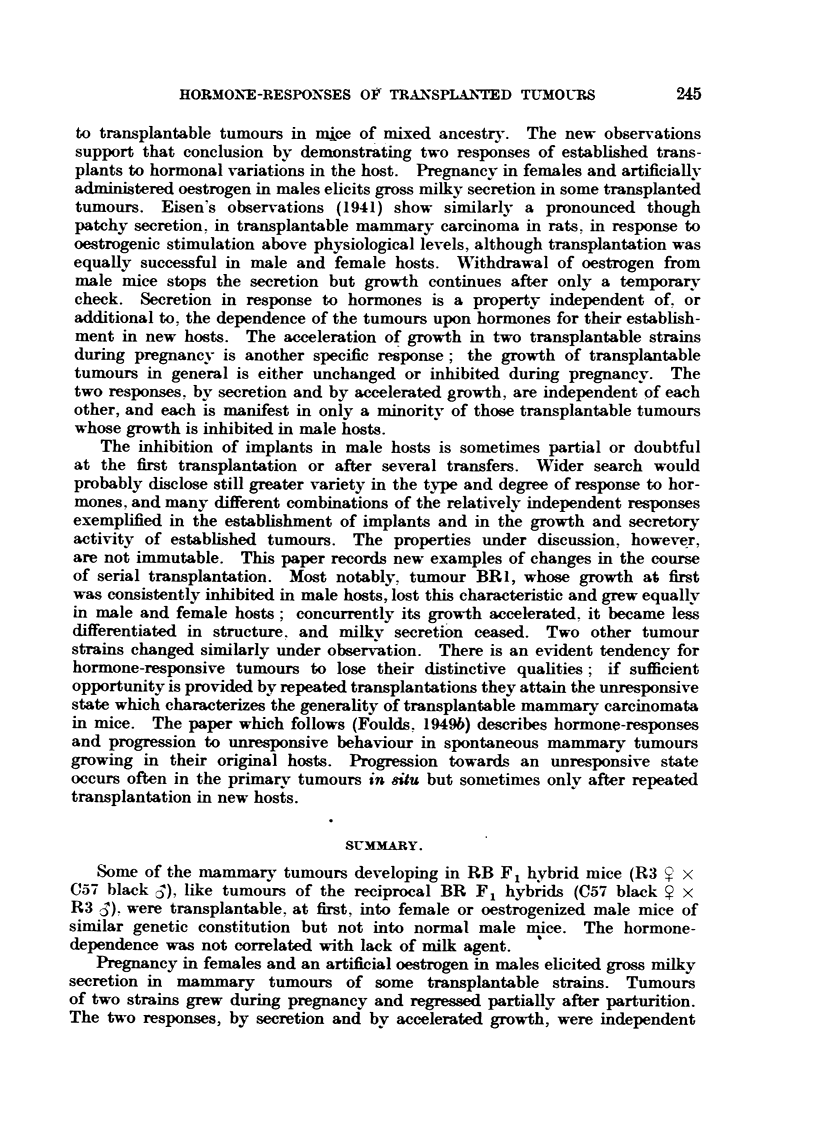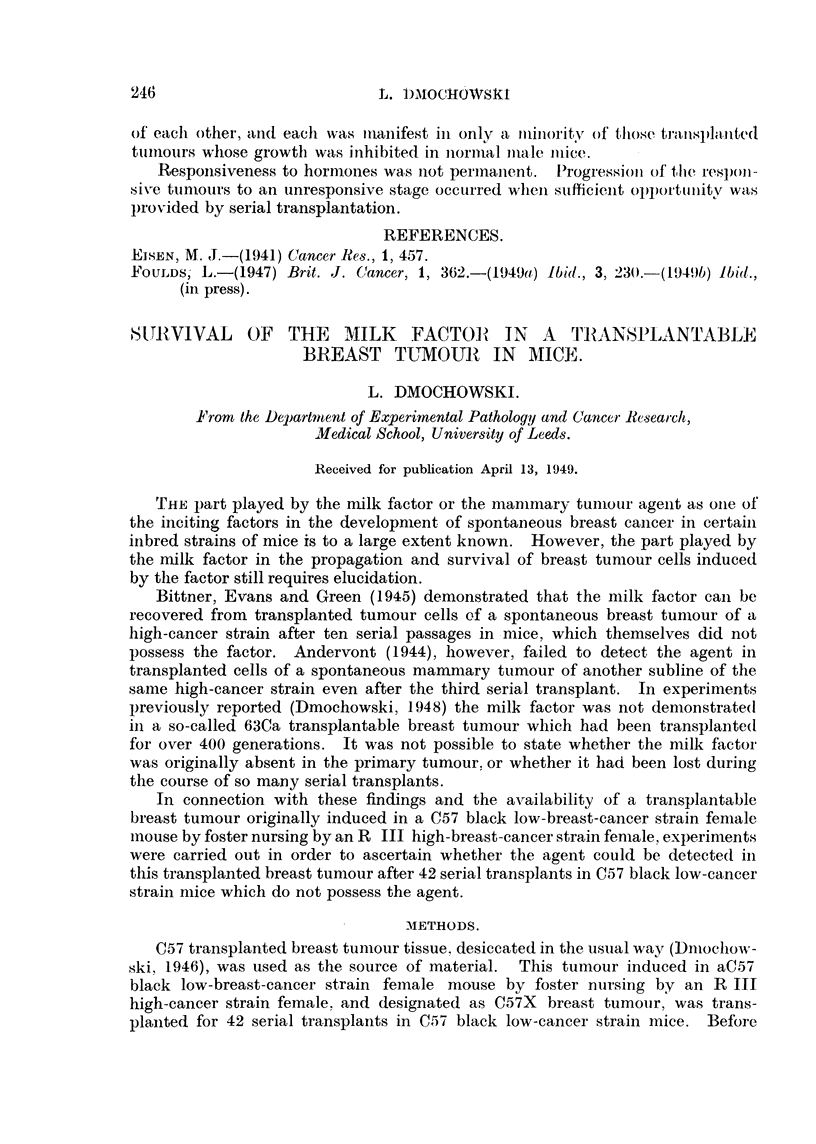# Mammary Tumours in Hybrid Mice: Hormone-Responses of Transplanted Tumours

**DOI:** 10.1038/bjc.1949.24

**Published:** 1949-06

**Authors:** L. Foulds


### MAMMARY TUMOURS IN HYBRID MICE: HORMONE-RESPONSES OF TRANSPLANTED TUMOURS.

L. FOULDS.

*From the Laboratories of the Imperial Cancer Research Fund, London, N.W.7.*

Received for publication March 14, 1949.

THIS paper records the later course of transplantable tumours already described (Foulds, 1947), and adds observations on the transplantation of tumours from other hybrids and on secretion in transplanted tumours. It provides evidence that hormone-responsiveness of transplanted tumours is not correlated with absence of the milk agent, that hormones regulate the activities of some transplanted tumours, and that responsiveness to hormones changes during serial transplantation.

### MATERIAL AND METHODS.

The earlier report described the origin and transplantation of tumours of $F_1$ mice of the cross C57 black ♀ × R3 ♂ (BR $F_1$), and an accompanying paper (Foulds, 1949*a*) recorded the incidence of tumours in the reciprocal hybrids RB $F_1$ (R3 ♀ × C57 black ♂), in BR hybrids of the $F_2$ to $F_5$ generations, and in BA $F_1$ hybrids (C57 black ♀ × A ♂) supplied artificially with milk agent. (For reasons given in the accompanying paper the designation of the C57 black ♀ × R3 ♂ hybrids has been changed from CR to BR. Tumours called CR1, CR2, etc., in the earlier report are here designated BR1, BR2, etc.)

Transplantation was carried out by the method used in the earlier experiments. The recipient mice were BR $F_1$ or RB $F_1$ hybrids, and each series included normal males as well as normal females or oestrogenized males or both. As a rule, transplantation was not continued beyond the second passage.

### *Transplantation of tumours of RB $F_1$ hybrids.*

The RB $F_1$ mice had the same genetic constitution as the BR $F_1$ mice used previously, but were nursed by their mothers of the high mammary tumour strain R3.

Twenty-five mammary tumours originating in 11 RB $F_1$ females were used for transplantation. Transplantation of 3 tumours was unsuccessful. Seven tumours from 5 mice grew in female or in oestrogenized male hosts, but grew tardily or not at all, within an observation period of 3 months or longer, in normal males. Implants of 15 tumours from 11 mice grew without conspicuous difference in male and female hosts; 4 of them were retarded slightly or inconstantly in males.

### *Transplantation of tumours of other hybrids.*

Tumours from hybrids other than BR $F_1$ and RB $F_1$ were not examined systematically. The limited observations showed that 4 out of 7 tumours of

BR $F_3$ hybrids were transplantable in female but not in male hosts, and that 1 of 4 tumours from BA $F_1$ hybrids was pártially and inconstantly inhibited in male mice.

*Changes in tumours during serial transplantation.*

The tumour BR1 was described in detail in the earlier paper as the prototype of hormone-responsive transplanted tumours. Serial transplantation was

FIG. 1.—Transplantation of tumour BR1. Solid squares denote growth in females or oestrogenized males ; solid circles growth in normal males and clear circles strong or complete inhibition of growth in males, less pronounced inhibition being represented by half-solid circles. The figures adjoining the vertical lines are the times, in days, between successive transplantations. Asterisks indicate the series in which milky secretion was conspicuous.

continued from 2 tumours of the second passage (Fig. 1). In one line of descent growth was consistently inhibited in male mice as far as the sixth passage. Two tumours of the sixth generation were transplanted about 12 months and 18 months respectively after the transplantation of the original BR1 tumour; implants of the first of these tumours grew after moderate delay in males as well as in females, and implants of the second grew equally in males and females.

Transplantation of this line was then stopped. Tumours in the other line of descent behaved similarly as far as the fourth passage. Three tumours of the fourth generation were transplanted. Implants of two of the tumours grew in female mice but not in males. The third tumour, transmitted by a large dose of minced tissue, grew in normal and in ovariectomized females and with only moderate delay in normal males. The tumour originated a distinct sub-strain of BR1 ; the tumours grew with equal facility in male and female hosts, they grew more rapidly than tumours of the original strain, they were less differentiated

FIG. 2.—Three successive generations of tumour BR1 showing increase in rate of growth and loss of sex-difference.

histologically, and they were not milky in contrast with many tumours of earlier passages as described in the section which follows. These characteristics are maintained in the fifteenth and current passage. In the eleventh passage implants grew in each of 3 males but in neither of 2 sibling females. The anomalous result was not repeated and was unexplained. Fig. 2 illustrates the contrast between tumours of 3 successive passages. The intervals between successive transplantations as shown in Fig. 1 give a rough indication of the acceleration of growth in the subline that is being continued.

The earlier paper recorded transplantation of 2 tumours from mouse BR6. The second and larger tumour BR6B grew from the outset equally in males and females, and maintained this behaviour up to the fifth generation, when transplantation was stopped. The earlier tumour was maintained in two lines of descent from the second generation onwards ; the tumours of one line grew only in female hosts, whereas tumours of the other line grew also with inconspicuous delay in some males. The two lines were continued to the ninth and tenth generations respectively. In the later passages they were less clearly differentiated. In the eighth and ninth passages tumours of the first line grew in males as well as in females. Tumours of the second line continued to grow in males but, sometimes, only after long delay. The two lines thus converged without abrupt changes in behaviour.

One other BR tumour (BR4) grew only in females in the first three passages and in the fourth grew equally in males and females. One tumour from a BA $F_1$ hybrid behaved erratically during 4 transfers, after which transplantation was discontinued ; in some series growth in females was superior to growth in males, but in other series no difference was apparent. The other transplantable tumours described in this and in the earlier paper were not carried through more than 2 or 3 passages.

*Secretion in transplanted tumours.*

In accordance with the general experience, histological examination disclosed varied degrees of secretory activity. Some BR1 tumours of the fourth to seventh transplanted generations, however, were conspicuously milky to gross inspection. The tumours were removed from mice in late stages of pregnancy, or from male mice carrying stilboestrol pellets ; tumours from nursing females were not examined. They oozed milky fluid when cut ; some contained small or large cysts with milky or creamy fluid ; some discharged similar fluid through ulcerated patches of skin. When slices of tumours were fixed in formol saline the secretory tumours were evident to cursory inspection by reason of the milkiness of the fixative. Histological examination showed accumulations of secretion, patchy in distribution ; tumour acini distended with secretion were contiguous with small, empty acini.

In the fifth passage of BR1, 23 male mice carrying stilboestrol pellets received implants. All the implants grew. Seven weeks after the transplantation the pellets were removed from nine mice. As previously recorded, removal of the pellets checked the growth of tumours temporarily, but no tumour retrogressed and soon all grew again at the customary rate. Three mice deprived of pellets died and were eaten by the survivors ; the remaining 6 mice were killed 7 to 12 weeks after removal of the pellets. None of the tumours was milky. Mice which retained their pellets were killed during the same period. Tumours from 12 out of 14 mice were milky in greater or less degree ; in one of the two mice with non-milky tumours the stilboestrol pellet was not found post mortem and evidence of oestrogenic action on the sex organs was lacking. Secretion was thus dependent on hormonal stimulation, whereas at that time growth was not so dependent.

Fig. 1 indicates (by asterisks) the passages in which conspicuously milky tumours were found. Examples were possibly overlooked or unrecorded in the first three passages. Thereafter milkiness was evident in pregnant females or in oestrogenized males in the series in which growth was inhibited in normal males.

None of the tumours in the later generations, when growth was rapid in normal males as well as in females, was milky, nor was any tumour of the other transplantable BR $F_1$ strains. Conspicuous milkiness was seen in the first passage of one BR $F_3$ tumour in oestrogenized males, and in one tumour from a mouse killed in late pregnancy in the second passage of a BA $F_1$ tumour. Two growths in the first passage of a RB $F_1$ tumour (BR10) were milky. Other transplanted RB tumours were not milky. Milky secretion evident to the naked eye occurred, therefore, in only a minority of those tumours whose transplantation was conditioned by hormones, and only under the stimulus of pregnancy or of an artificial oestrogen.

*Response of transplanted tumours to pregnancy.*

The transplanted tumours of two RB strains (RB9 and RB10) and one BR $F_4$ strain (BR $F_4$ 90) which grew only in females increased in size whilst their hosts were pregnant and diminished after parturition. The regression was considerable but not complete (Fig. 3). The effect persisted in the second passage

FIG. 3.—Three tumours selected from first and second transplanted generations of RB10. Pregnancy recorded at the weekly chartings marked " P."

of RB 10, which was not further transplanted, and in the second and third (current) passage of RB9. This pregnancy response was not observed in other transplanted BR or RB tumours, including those which secreted during pregnancy.

### DISCUSSION.

At the time of the earlier report (Foulds, 1947) no information about the presence of mammary tumour agent or " milk agent " in BR $F_1$ mice and their tumours was available, but subsequent observations (Foulds, 1949*a*) provided evidence of the presence of tumour agent in transplanted tumours of the BR1 strain and in the BR $F_1$ mice from which derived two other tumours (BR4 and BR6) whose transplantation was conditioned by the sex of the host. It is now shown that mice of the reciprocal cross RB $F_1$ which receive tumour agent from their high mammary tumour strain mothers develop mammary tumours whose transplantation is similarly conditioned. It is evident that the dependence of some transplantable mammary tumours on appropriate hormonal conditions is not correlated with a deficiency of the milk agent.

The earlier report attributed the inhibition of implants in normal male hosts to lack of an essential stimulus which was provided by normal females and by oestrogenized males ; it discounted the operation of the well-known resistance to transplantable tumours in mice of mixed ancestry. The new observations support that conclusion by demonstrating two responses of established transplants to hormonal variations in the host. Pregnancy in females and artificially administered oestrogen in males elicits gross milky secretion in some transplanted tumours. Eisen's observations (1941) show similarly a pronounced though patchy secretion, in transplantable mammary carcinoma in rats, in response to oestrogenic stimulation above physiological levels, although transplantation was equally successful in male and female hosts. Withdrawal of oestrogen from male mice stops the secretion but growth continues after only a temporary check. Secretion in response to hormones is a property independent of, or additional to, the dependence of the tumours upon hormones for their establishment in new hosts. The acceleration of growth in two transplantable strains during pregnancy is another specific response; the growth of transplantable tumours in general is either unchanged or inhibited during pregnancy. The two responses, by secretion and by accelerated growth, are independent of each other, and each is manifest in only a minority of those transplantable tumours whose growth is inhibited in male hosts.

The inhibition of implants in male hosts is sometimes partial or doubtful at the first transplantation or after several transfers. Wider search would probably disclose still greater variety in the type and degree of response to hormones, and many different combinations of the relatively independent responses exemplified in the establishment of implants and in the growth and secretory activity of established tumours. The properties under discussion, however, are not immutable. This paper records new examples of changes in the course of serial transplantation. Most notably, tumour BR1, whose growth at first was consistently inhibited in male hosts, lost this characteristic and grew equally in male and female hosts; concurrently its growth accelerated, it became less differentiated in structure, and milky secretion ceased. Two other tumour strains changed similarly under observation. There is an evident tendency for hormone-responsive tumours to lose their distinctive qualities; if sufficient opportunity is provided by repeated transplantations they attain the unresponsive state which characterizes the generality of transplantable mammary carcinomata in mice. The paper which follows (Foulds, 1949*b*) describes hormone-responses and progression to unresponsive behaviour in spontaneous mammary tumours growing in their original hosts. Progression towards an unresponsive state occurs often in the primary tumours *in situ* but sometimes only after repeated transplantation in new hosts.

### SUMMARY.

Some of the mammary tumours developing in RB $F_1$ hybrid mice (R3 ♀ × C57 black ♂), like tumours of the reciprocal BR $F_1$ hybrids (C57 black ♀ × R3 ♂), were transplantable, at first, into female or oestrogenized male mice of similar genetic constitution but not into normal male mice. The hormone-dependence was not correlated with lack of milk agent.

Pregnancy in females and an artificial oestrogen in males elicited gross milky secretion in mammary tumours of some transplantable strains. Tumours of two strains grew during pregnancy and regressed partially after parturition. The two responses, by secretion and by accelerated growth, were independent of each other, and each was manifest in only a minority of those transplanted tumours whose growth was inhibited in normal male mice.

Responsiveness to hormones was not permanent. Progression of the responsive tumours to an unresponsive stage occurred when sufficient opportunity was provided by serial transplantation.

### REFERENCES.

Eisen, M. J.—(1941) *Cancer Res.*, **1**, 457.

Foulds, L.—(1947) *Brit. J. Cancer*, **1**, 362.—(1949*a*) *Ibid.*, **3**, 230.—(1949*b*) *Ibid.*, (in press).